# Supplementary material for: Report of a Novel Molecular Profile in Malignant Insulinoma
Source: J Clin Med. 2023 Feb 6;12(4):1280. doi: 10.3390/jcm12041280 (PMC9962228; doi:10.3390/jcm12041280)
Supplement: Supplementary file 1 [file jcm-12-01280-s001.zip › Supplementary file 2_Insulinoma Clinical Trials 110622.pdf]

ClinicalTrials.gov Search Results 11/06/2022

	Title	Status	Study Results	Conditions	Interventions	Locations
1	The Clinical Application of 68Ga-NOTA-exendin-4 PET/CT in Detecting Insulinoma	Recruiting	No Results Available	•Insulinoma	•Drug: 68Ga-NOTA-exendin-4	•Department of Nuclear Medicine, First Affiliated Hospital of Fujian Medical University, Fuzhou, Fujian, China
2	[68Ga]Ga-HBED-CC-exendin-4 and [68Ga]Ga-NOTA-exendin-4 PET/CT Imaging in the Same Group of Insulinoma Patients	Active, not recruiting	No Results Available	•Insulinoma	•Drug: [68Ga]Ga-HBED-CC-exendin-4<br>•Drug: [68Ga]Ga-NOTA-exendin-4	•Peking Union Medical College Hospital, Beijing, Beijing, China
3	A Multi-center Study to Evaluate the Efficacy and Safety of Pancreatic Duct Stents Placement Before the Enucleation of Insulinoma Located in the Head and Neck of the Pancreas Near the Main Pancreatic Duct	Not yet recruiting	No Results Available	•Insulinoma	•Procedure: placement of pancreatic duct stents before enucleation surgery<br>•Procedure: Direct enucleation surgery	•Peking Union Medical College Hospital, Beijing, Beijing, China
4	EUS-guided Ethanol Ablation of an Insulinoma	Unknown status	No Results Available	•Insulinoma	•Procedure: EUS-guided ethanol ablation	•First Affiliated Hospital of Guangxi Medical University, Nanning, Guangxi, China
5	Clinical Evaluation of 68Ga-NOTA-MAL-Cys39-exendin-4 Positron Emission Tomography in the Detection of Insulinoma	Unknown status	No Results Available	•Insulinoma	•Drug: 68Ga-NOTA-MAL-Cys39-exendin-4	•Nanjing First Hospital, Nanjing, Jiangsu, China
6	68Ga-NOTA-exendin-4 PET/CT for the Localization of Insulinoma and Diagnosis of Nesidioblastosis	Unknown status	No Results Available	•Insulinoma<br>•Nesidioblastosis	•Drug: 68Ga-NOTA-exendin-4	•Peking Union Medical College Hospital, Beijing, Beijing, China
7	Fluorodopa F 18 in Congenital Hyperinsulinism and Insulinoma	Recruiting	No Results Available	•Congenital Hyperinsulinism<br>•Insulinoma	•Drug: Fluorodopa F 18	•Cook Children's Medical Center, Fort Worth, Texas, United States
8	Application of Raw Corn Starch on Patients With Insulinoma	Unknown status	No Results Available	•Hyperinsulinemic Hypoglycemia<br>•Insulinoma<br>•Raw Corn Starch	•Other: low GI diet with regular supplementation of raw corn starch	•Peking Union Medical College Hospital, Beijing, China
9	Diagnosing and Treating Low Blood Sugar Levels	Recruiting	No Results Available	•Hypoglycemia<br>•Insulinoma		•National Institutes of Health Clinical Center, Bethesda, Maryland, United States
10	CGM (Continuous Glucose Monitoring) Use in Diagnosis of Spontaneous and Reactive Hypoglycaemia	Unknown status	No Results Available	•Hypoglycemia, Reactive<br>•Hypoglycaemia Night<br>•Hypoglycemia Non-Diabetic<br>•Hypoglycemia Unawareness<br>•Insulinoma<br>•Insulinoma; Malignant, Pancreas<br>•Insulin Hypoglycemia<br>•Insulin Resistance<br>•Spontaneous Hypoglycemia<br>•Neuro Endocrine Tumours	•Device: use of continuous glucose monitoring	•St Bartholomew's Hospital, dept of endocrinology, London, United Kingdom
11	177Lu-DOTATOC for the Treatment of Patients With Somatostatin Receptor Positive NETs	Not yet recruiting	No Results Available	•Neuroendocrine Tumors<br>•Carcinoid Tumor<br>•Pulmonary Carcinoid Tumor<br>•Gastroenteropancreatic Neuroendocrine Tumor<br>•Vipoma<br>•Insulinoma<br>•Gastrinoma	•Drug: 177Lu-DOTATOC	•BC Cancer, Vancouver, British Columbia, Canada

	Title	Status	Study Results	Conditions	Interventions	Locations
12	DOTATOC PET/CT for Imaging NET Patients	Recruiting	No Results Available	•Neuroendocrine Tumors<br>•Insulinoma<br>•Gastrinoma<br>•Glucagonoma<br>•Vipoma<br>•Pheochromocytoma<br>•Paraganglioma<br>•Neuroblastoma<br>•Ganglioneuroma<br>•Medullary Carcinoma<br>•and 6 more	•Diagnostic Test: 68Ga-DOTATOC PET/CT<br>•Diagnostic Test: 18F-FDG PET/CT	•BC Cancer, Vancouver, British Columbia, Canada
13	Temozolomide With or Without Capecitabine in Treating Patients With Advanced Pancreatic Neuroendocrine Tumors	Active, not recruiting	Has Results	•Gastrinoma<br>•Glucagonoma<br>•Insulinoma<br>•Islet Cell Carcinoma<br>•Pancreatic Polypeptide Tumor<br>•Recurrent Islet Cell Carcinoma<br>•Somatostatinoma	•Drug: temozolomide<br>•Drug: capecitabine	•Kaiser Anaheim Medical Center, Anaheim, California, United States<br>•Kaiser Permanente-Deer Valley Medical Center, Antioch, California, United States<br>•Kaiser Permanente Medical Group - Baldwin Park, Baldwin Park, California, United States<br>•Kaiser Foundation Hospital, Bellflower, California, United States<br>•Alta Bates Summit Medical Center-Herrick Campus, Berkeley, California, United States<br>•Mills - Peninsula Hospitals, Burlingame, California, United States<br>•Kaiser Permanente Hospital, Fontana, California, United States<br>•Kaiser Permanente, Fremont, Fremont, California, United States<br>•Kaiser Permanente, Fresno, California, United States<br>•Kaiser Permanente - Harbor City, Harbor City, California, United States<br>•and 313 more
14	Utility of 18FDOPA PET/MRI for Focal Hyperinsulinism	Recruiting	No Results Available	•Hyperinsulinism	•Drug: 18F-Fluoro Dopa PET/MRI Imaging	•Washington University, Saint Louis, Missouri, United States

	Title	Status	Study Results	Conditions	Interventions	Locations
15	Disease-Specific Questionnaire in Assessing Quality of Life in Patients With Gastrointestinal-Related Neuroendocrine Tumors	Unknown status	No Results Available	•Gastrointestinal Carcinoid Tumor<br>•Islet Cell Tumor<br>•Lung Cancer<br>•Metastatic Cancer<br>•Neoplastic Syndrome	•Other: questionnaire administration<br>•Procedure: quality-of-life assessment	•Aarhus Universitetshospital - Aarhus Sygehus, Aarhus, Denmark<br>•Charite - Campus Charite Mitte, Berlin, Germany<br>•Klinikum der Universitaet Regensburg, Regensburg, Germany<br>•University Athens Alexandras Hospital, Athens, Greece<br>•Hadassah University Hospital, Jerusalem, Israel<br>•Clinical and Experimental Medicine at the University of Verona, Verona, Italy<br>•Netherlands Cancer Institute - Antoni van Leeuwenhoek Hospital, Amsterdam, Netherlands<br>•Maria Sklodowska-Curie Memorial Cancer Center and Institute of Oncology - Warsaw, Warsaw, Poland<br>•Hospital Universitario San Carlos, Madrid, Spain<br>•Uppsala University Hospital, Uppsala, Sweden<br>•and 6 more

U.S. National Library of Medicine | U.S. National Institutes of Health | U.S. Department of Health & Human Services
